# The Toxicological Aspects of the Heat-Borne Toxicant 5-Hydroxymethylfurfural in Animals: A Review

**DOI:** 10.3390/molecules25081941

**Published:** 2020-04-22

**Authors:** Mayada R. Farag, Mahmoud Alagawany, May Bin-Jumah, Sarah I. Othman, Asmaa F. Khafaga, Hazem M. Shaheen, Dalia Samak, Abdelrazeq M. Shehata, Ahmed A. Allam, Mohamed E. Abd El-Hack

**Affiliations:** 1 Forensic Medicine and Toxicology Department, Veterinary Medicine Faculty, Zagazig University, Zagazig 44519, Egypt; 2 Poultry Department, Faculty of Agriculture, Zagazig University, Zagazig 44519, Egypt; dr.mahmoud.alagwany@gmail.com; 3 Biology Department, College of Science, Princess Nourah bint Abdulrahman University, Riyadh 11671, Saudi Arabia; may_binjumah@outlook.com (M.B.-J.); sialothman@pnu.edu.sa (S.I.O.); 4 Department of Pathology, Faculty of Veterinary Medicine, Alexandria University, Edfina 22758, Egypt; Asmaa.Khafaga@alexu.edu.eg; 5 Department of Pharmacology, Faculty of Veterinary Medicine, Damanhour University, Damanhour 22511, Egypt; dr_hazemshaheen3010@yahoo.com; 6 Department of Veterinary Forensic Medicine and Toxicology, Faculty of Veterinary Medicine, Damanhour University, Damanhour 22511, Egypt; dalia_samak@vetmed.dmu.edu.eg; 7 Department of Animal Production, Faculty of Agriculture, Al-Azhar University, Cairo 11651, Egypt; abdelrazeq@azhar.edu.eg; 8 Department of Dairy Science & Food Technology, Institute of Agricultural Sciences, Banaras Hindu University, Varanasi 221005, India; 9 Department of Zoology, Faculty of Science, Beni-suef University, Beni-suef 65211, Egypt; allam1081981@yahoo.com

**Keywords:** toxicology, heat-borne, Maillard reaction, 5-hydroxymethylfurfural, toxicant

## Abstract

The incidence of adverse reactions in food is very low, however, some food products contain toxins formed naturally due to their handling, processing and storage conditions. 5-(Hydroxymethyl)-2-furfural (HMF) can be formed by hydrogenation of sugar substances in some of manufactured foodstuffs and honey under elevated temperatures and reduced pH conditions following Maillard reactions. In previous studies, it was found that HMF was responsible for harmful (mutagenic, genotoxic, cytotoxic and enzyme inhibitory) effects on human health. HMF occurs in a wide variety of food products like dried fruit, juice, caramel products, coffee, bakery, malt and vinegar. The formation of HMF is not only an indicator of food storage conditions and quality, but HMF could also be used as an indicator of the potential occurrence of contamination during heat-processing of some food products such as coffee, milk, honey and processed fruits. This review focuses on HMF formation and summarizes the adverse effects of HMF on human health.

## 1. Introduction

During food processing (cooking/heat treatment), several substances are formed. Some of these substances may add taste, color and texture while minimizing harmful germs. Some newly-formed compounds may have antimicrobial, antiallergenic and antioxidant activity [1]. Besides these beneficial substances, some other substances should be cautiously evaluated.

5-(Hydroxymethyl)-2-furfural (HMF), a heat-induced food toxicant, is formed in many food items, and causes several adverse effects in animals. HMF has been found in a wide variety of food products like dried fruit, fruit juice, caramel products, coffee, bakery products, malt and vinegar [2,3]. It has been also detected in cigarette smoke and chewing tobacco [4,5]. The toxicological effects of HMF have been demonstrated in several experimental animals and in cultured mammalian cells [6,7,8,9,10]. The daily human intake of HMF was reviewed by Husøy et al. [11] and ranged from 30 and 150 mg/person/day, a dose much higher than that of other food heat-borne toxicants like furan and acrylamide [12,13]. An estimation by the Federal Institute of Risk Assessment [14] showed a range from 4 to 30 mg HMF/person/day.

Cytotoxic, genotoxic, mutagenic and carcinogenic activities of HMF in rats and mice have been reported earlier [9,15]. Lee et al. [16] reported that HMF is an indirect bacterial mutagen due to its active metabolite, sulfuric acid ester 5-sulfo-oxymethylfurfural (SMF). The metabolic formation of SMF was illustrated by activated mutagenicity of HMF in the presence of rat hepatic cytosol enriched with the sulfogroup donor, 3′-phosphoadenosine-5′-phosphosulfate (PAPS). SMF was found to act as a mutagen in mammalian cultured cells and to initiate tumours in mouse skin [17]. Sulfur conjugation has been found to play a central role in metabolic activation, mutagenicity, and carcinogenicity of several environmental toxicants that include aromatic amines, alkenylbenzenes, and polynuclear aromatic hydrocarbons [6], indicating that the toxic effects of HMF can be metabolically enhanced via some of its metabolites.

Because of the wide distribution, high dietary exposure and toxic potential of HMF and the lack of reliable mitigation procedures to decrease its load in food products, natural dietary components should be investigated as alternative strategies to prepare the body to counteract the hazardous impacts of such contaminants.

## 2. Definition of 5-Hydroxymethylfurfural (HMF)

5-(Hydroxymethyl)-2-furfural (HMF), represents a wide class of heterocycles and is formed as an intermediary product of the Maillard reaction [18] or formed by carbohydrate dehydration in an acid medium. HMF also can be generated in significantly amounts at low temperatures during long periods of storage [19]. The formation of HMF is affected by the concentration and type of sugar, acid, minerals, pH as well as amino acids.

In a low pH environment HMF could be formed at low-temperature levels [20], and its values, were greatly increased as the temperature levels of either thermal manipulations or the storage circumstances increased. The alternative routes of dehydration and pyrolysis could be mentioned as an irregular way to generate HMF from either sucrose or fructose. This is associated with the generation of an extremely reactive fructofuranosyl cation which could be efficient and clearly converted into HMF under dry conditions [21].

## 3. Occurrence and Dietary Exposure to HMF

**Figure 1:**
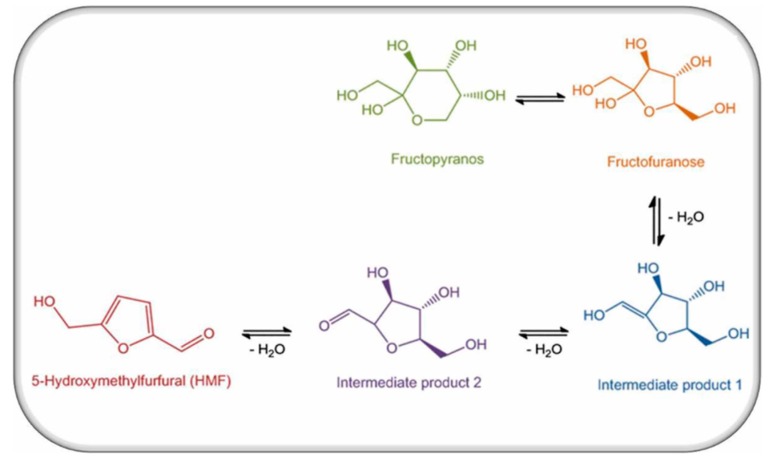
Production of HMF in honey.

The level of measurable HMF in food materials is clearly associated with the heat of reaction. The formation of HMF is an essential marker of temperature changes during storage in different food products such as juice and honey [22]. HMF can be formed in food products via different routes such as acid catalysed degradation of reducing sugars or through Maillard reactions. The formation of HMF is not only an indicator of the storage conditions and food quality, but also indicates the potential of occurrence of contamination during Maillard reactions, or by dehydration [23]. In addition to the presence of HMF in honey (Figure 1) and in preserved fruits (> 1 g/kg), it also occurs in other food ingredients including instant coffee or caramel (more than 6.2 g/kg), milk, citrus juices, apple juice, baked foods, breakfast cereal, and tomato byproducts. HMF is originated from sugars during food processing. Humans consume many different kinds of food products which are usually subjected to thermal manipulation steps such as boiling, pasteurization, roasting or baking before their consumption,. Throughout the heat course or when preparing food for storage, the Maillard reaction could occur and HMF be formed according to either the processing or storage conditions [24].

**Figure 2:**
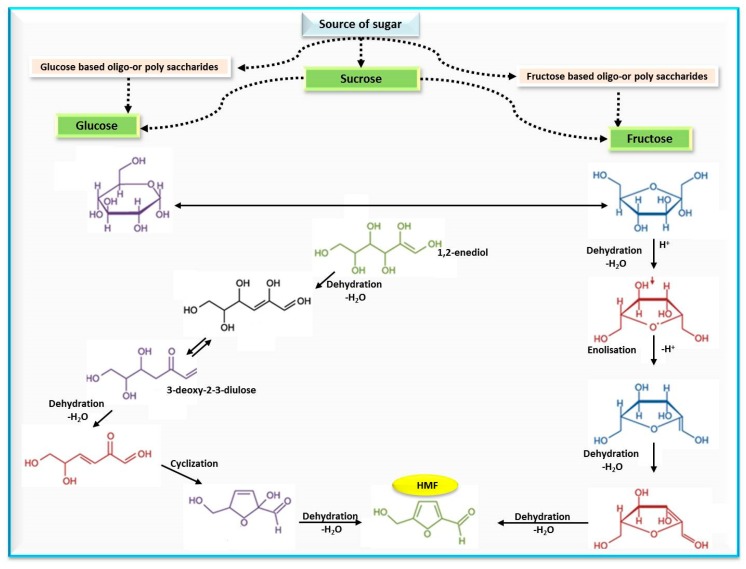
Mechanism of HMF formation from simple sugars.

Polovková and Šimko [23] detected HMF levels by high performance liquid chromatography supported with a diode array detector (HPLC-DAD) at 284 nm, and their findings showed the presence of HMF in 25 kinds of brown sugar, 15 kinds of which had HMF values that ranged from 0.17 to 6.45 mg/kg, while 13 kinds of white sugar had zero HMF. The authors attributed the occurrence of HMF in brown sugar to a lack of refining or as a result of adding treacle during the production process. These results were compatible with a previous study, where values of HMF in sugar-containing products were estimated and recorded (10.9–16.4, and 12.3–23.3 mg/kg) for light- and dark-brown sugars, respectively. The mechanism of HMF formation from simple sugars is illustrated in Figure 2.

Mańkowska et al. [25] reported that distinct HMF levels could be found in 41 kinds of food. Wheat bread with cranberries displayed the highest HMF level (210 mg/kg), breakfast cereals, (i.e., honey wheat loops; 85.09 mg/kg), while depleted values of HMF were found in gluten-free sponge cakes or whole-grain oatmeal. Sweetened breakfast cereals included 25.55 mg/kg HMF, 39% higher than the average content in bakery food products (18.40 mg/kg). Moreover, the HMF average values in cereals, (i.e., combined grains (240 mg/kg), cornflakes (7–114 mg/kg) and wheat-associated cereals (6–132 mg/kg) were also high [26,27]. Food products that undergo fermentation and some types of flour are usually used in bread and other bakery products. HMF values in bread manufactured from rye flour was recorded to be the highest level (average 26.88 mg/kg) and could be attributed to the high amino acid composition.

Also, some kinds of preserved fruits such as apple, strawberries, raisins, palms, cranberries, and red currants normally have great levels of HMF. HMF contents in rice-wheat flakes were determined to range from 6.78 to 11.70 mg/kg, while a lower level of HMF (6.06 mg/kg) was detected in by-products including raisins or plums. However, red fruits such as apples, red currants and strawberries have the highest levels of HMF [25]. Hence, white bread with preserved fruits has higher HMF levels than white bread alone [28,29]. Coffee, a common drink, also contains HMF and its concentration depends on the kind of coffee (plunger-brewed coffees, filtered coffee, mocha or espresso) and amount of sugar added. Mortas et al. [30] determined HMF in Turkish coffees (either instant brand or traditionally prepared) by HPLC supported with a diode array indicator. They reported that prior to steeping, instant and traditional Turkish coffee specimens contain HMF levels of 213.02–238.99 and 336.03–362.05 mg/kg, respectively. After preparation, the HMF content was elevated in instant coffee by 32.29–55.83%, while the concentration of HMF in traditional coffee increased by 74.12–224.75%.

Arribas-Lorenzo and Morales [31] used reversed-phase chromatography supported by UV-detection to estimate HMF concentrations in three kinds of ground coffee consumed by the Spanish population and observed clear variations in the contents. HMF values were 110 mg/kg (original coffee: manufactured by ordinary roasting of coffee beans), 625 mg/kg (torrefacto coffee: manufactured by mixing sucrose before the roasting step) and 1734 mg/kg (blended coffee: a mixture of original and ground torrefacto coffee in different ratios), while soluble coffee had the highest value (2480 mg/kg). The authors showed that the HMF consumption along with heavy coffee adult consumers in Spain was about 122.42 μg/kg/day, and suggesting an important thermal contribution to HMF generation.

HMF can be formed in dairy products by side chemical reactions during thermal sterilization and browning processes [32]. Indeed, the Maillard reaction is undesirable in infant milk because these the precursors might be the only source of lysine for babies [33]. Identical HPLC conditions were used to measure free and total HMF in liquid and powdered infant milk formulas to estimate the effect of the length and temperature of storage on the extent of the Maillard reaction in infant milk. A linear increase in the concentration of free and total HMF was found with increasing storage time and temperature in both powdered and liquid infant milk, however, values of total and free HMF were higher in powdered infant milk in comparison with the liquid milk of the same commercial brand. These differences may due to the temperature treatments applied to powdered milk during the manufacturing process [34]. There were no significant differences in total HMF content between milk stored at 4 °C or those stored at 8 °C, while storage this milk for 24 weeks caused a significant increase in the total HMF content by about 1.7 fold compared to the value estimated directly after production. However, storing the milk for the same period (24 weeks) at room temperature showed a 2.06-fold increase in HMF content [35]. These findings also indicate that both storage temperature and storage time can increase the HMF content in the UHT sterilised milk.

Fruits and vegetables are rich in amino acids and sugars which increases the potential for HMF formation. In a previous study, jam was stored at 20 or 35 °C for one year, and temperature and storage duration were found to be involved in an increased HMF generation [2]. Furthermore, a firm relationship between storage duration and thermal environmental conditions along with HMF generation has been reported for two kinds of apple juice [2]. Ordóñez-Santos et al. [36] observed the variations in HMF values in bottled tomato pulp product stored for 180 days at 20 °C. At the end of this trial, the concentration of HMF had increased by 152%, while a significant reduction in levels of organic acids (malic acid, ascorbic acid and citric acid) was found. The authors suggested a correlation between the fall in organic acid levels and HMF generation. These findings were similar to those of a previous study by Min and Zhang [37], who attributed the formation of non-enzymatic browning products in stored tomato juice to the reaction between amino acids and carbonyl groups released from ascorbic acid degradation.

In order to store some fruits for a long time, drying is one of the common methods used for this purpose. Formation of HMF in the different dried fruits depends mainly on their sugar and acid content. A comparative study of HMF formation potentials in some dried fruits with high sugar content that were subjected to heat processes for prolonged time was performed. The results showed a high variation among their potentials to form HMF. The lowest concentration of HMF was found in dried figs (1 mg/kg), while the highest concentration of HMF was found in dried plums (2200 mg/kg). Dried dates also showed a high level of HMF content (1000 mg/kg). Some other fruits such as pear, peach, apple and pineapple had significantly less HMF, which may be attributed to their composition or processing differences [38]. Indeed, the formation of HMF in drying plant tissue has only been found in fruits that contain a high concentration of sugar and subjected to heat-processing for long times. In this aspect, the formation of HMF in drying vegetables was not found in most of the examined samples such as cabbage, broccoli and artichoke, while samples of dehydrated vegetable extracts (artichoke, cabbage and tomato) showed a high concentration of HMF (6.97, 58.6 and 18.2, respectively). These findings may me due to heat treatments during percolation and/or atomisation processes [26].

As far as oil content is concerned, the oil level in commercial products could influence HMF formation. To explore this concept, the level of HMF was estimated in defatted and whole crushed hazelnuts after a roasting process to evaluate the effect of oil concentration on HMF formation. The results showed a critical role of oil as a stimulator of HMF generation. There was a high correlation between HMF levels and the oil concentration in hazelnuts roasted at 175 °C for 30 min. Interestingly, when sucrose was added to full-fat and defatted crushed hazelnuts before roasting at 175 °C for 30 min, HMF recorded the highest value (372.4 mg/kg) in full-fat hazelnuts sample, while a very low value (33.5 mg/kg) was observed in the defatted sample. These results confirmed the central role of oil concentration in roasted hazelnuts, as the HMF content increased from 66.5 to 144.0 mg/kg in non-defatted specimens exposed to extended dry heating (from 30 to 60 min, respectively) [39]. Monakhova and Lachenmeier [40] reported that HMF can be formed in most heat-treated foods with a variation in the levels ranged from trace concentration in juices to nearly 4,000 mg/kg in coffee.

## 4. Acceptable Daily Intake of HMF

The medium and high daily HMF consumption has been estimated at 5.26 mg and 8.57 mg respectively [41]. Most of this dietary exposure comes from coffee, which accounts for about 50% of the estimated total HMF consumption in Spain [42] and about 63% in Norway [11]. The estimated level of HMF exposure ranged from 30 to 150 mg per person [43]. Findings from earlier studies reported that the toxic effects of HMF were observed with a dose more than 75 mg/kg body weight [44]. Zaitsev et al. [45] reported 2 mg of HMF/kg body weight is suggested as an acceptable daily intake from food for human beings, while findings from other studies observed that daily consumption of HMF should range from 2–30 mg per person/day [41,46].

## 5. Uses of HMF

HMF can be widely used as an indicator of the quality of food products such as coffee [47], milk [32], honey [48] or processed fruits [2]. HMF was also used for checking the thermal procedures applied to commercial cereal products such as breakfast cereals [49], pasta preservation [50], bread slice toasting [51] or bread baking [28], as well as baby cereals [52]. Thermal processing of food plays a key role in improving the digestibility and absorption as well as the availability of bioactive compounds due to cell breakdown. However, aggressive and long high heat processing may lead to damage and loss of some bioactive compounds. It has been shown that HMF can be used as a useful marker to control heat processing time and the type and intensity of heat-treating of cooked vegetables. In addition, an association has been found between HMF and the antioxidant capacity potential of vegetables exposed to various cooking techniques [53].

## 6. Metabolism of HMF

It was demonstrated from oral gavage administration trials of [^14^C]-HMF at different dosages (0.08–500 mg/kg body weight) that HMF extensively passed through the digestive canal in both rats and mice [54]. In a Caco2 cell line, Delgado-Andrade et al. [55] reported that HMF absorption could be increased when cells were subjected to a higher HMF level. It has been demonstrated that HMF absorption can be affected also by food composition such as fiber content. Moreover, the bioavailability of HMF in three commercial breakfast cereals ranged from 4.98 to 12.99%. This variation may due to variations in the composition of each breakfast cereals, while fiber content also plays an essential role [55].

The main biotransformation pathway of HMF occurred through HMF oxidation to 5-hydroxymethyl-2-furanoic acid (HMFA) followed by glycine conjugation to form *N*-(5-hydroxymethyl-2-furoyl)glycine (HMFG) as the principal metabolite eliminated in the urine [41]. In rat and human trials, the HMFA/HMFG ratio dropped as the HMF dose increased, suggesting that confined glycine could minimize the conjugation reaction rate, leading to the elimination of either free furoic acid (FA) or 2,5-furandicarboxylic acid (FDCA) via other routes [56].

On the other hand, a small human study was conducted on seven adult persons, where the urine excretion of HMF was determined to estimate the residual HMF in the body. The participants consumed 20 g of plum jam contain 24 mg of HMF, and only 163 µg were detected in urine after 6 h, indicating that 99.25% of the consumed HMF remained in the body [3].

In addition, HMF has been also observed to be transformed in vivo into 5-sulfoxymethyfurfural (SMF) via sulphonation of its allylic hydroxyl active group initiated by sulfotransferases (SULTs) and the sulpho group donor 3-phosphoadenosine-5--phosphosulphate (PAPS). SMF is a unstable form, however, it has been detected in the bloodstream of HMF-treated mice and humans, indicating that HMF was transformed into SMF in an vivo model [7,46].

Some trials were conducted using [^14^C]-HMF. The results indicated that dietary HMF was obviously and clearly transformed and eliminated via urine or to a lower degree in hepatic tissue [54].

## 7. Toxicity of HMF

**Figure 3:**
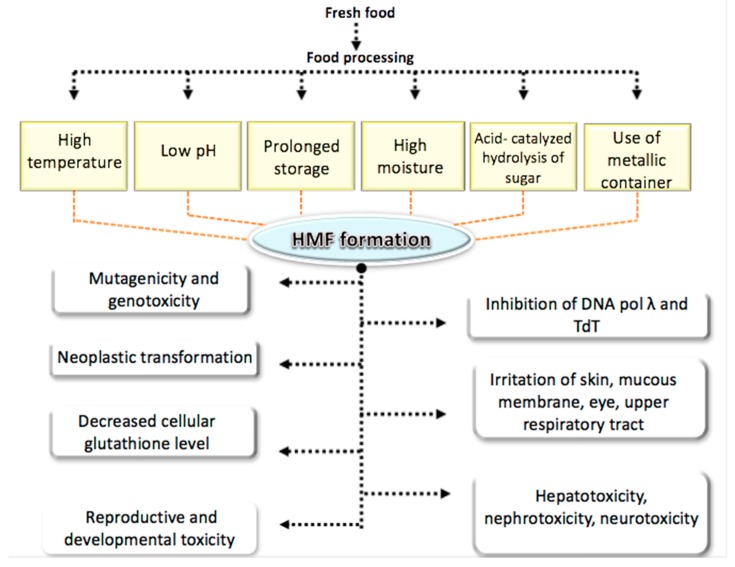
Adverse effect of HMF on human health.

Several studies have reported various adverse effects of HMF on human health as described in the following sections and summarized in Figure 3.

### 7.1. Hepatotoxic Effects

Previous toxicity trials of HMF stated that oral LD_50_ values were recorded to be 1910 and 3100 mg/kg for mouse and rats models, respectively [41]. Daily oral HMF consumption of 310 mg/kg for 60 days could cause an impairment of liver functions (an alteration in the serum protein nd albumin to globulin ratio), and a hepatic tributyrinase in rats. In addition, enterokinase activity was increased in response to this treatment [44]. It has been reported that consumption of 188 or 375 mg/kg HMF for about two years could elevate the incidence of hepatocellular adenomas in B6C3F1 female mice, indicating a carcinogenic effect of exposure to high HMF levels for a long time [57].

Bauer-Marinovic et al. [58] observed that administration of 250 mg/kg SMF to FVB/N mice, caused damage to the tubules and only moderate toxicity to the liver. Following HMF consumption, it is transformed into SMF in the liver and then released into the bloodstream to induce toxicity in other tissues, while uptake of SMF into the liver is required to evoke hepatotoxicity.

### 7.2. Nephrotoxic Effect

Some chemical substances can be activated metabolically to form carcinogens and mutagens. SMF is an electrophilic metabolite of HMF. The strong nephrotoxic effects of SMF were demonstrated from several studies [7,58]. These nephrotoxic effects usually target the proximal tubules and cause a great deterioration in them. Interestingly, HMF is rapidly absorbed in the gastrointestinal tract. Large amounts of HMF metabolites can be excreted via urine [56], however, among these metabolites, SMF, the most significant ultimate toxicant, is non-excretable [32]. Supplementation of HMF at high doses in drinking water for three months resulted in mild toxic effects in mice, particularly in the kidneys [58]. Findings from previous studies indicated that SMF administration has the potential to cause nephrotoxicity, but SMF transformed from HMF does not appear to have the same potential [58], although humans are not directly exposed to SMF.

Evidence from previous molecular studies reported that uptake, accumulation and toxicity of SMF in renal cells which caused damage to proximal tubules was mediated by organic anion transporters (OAT1 and OAT3). The authors reported that the cytotoxicity in hOAT1- and hOAT3-expressing cells showed a significant increase in comparison with control cells, while this cytotoxicity effect of SMF was decreased following addition an inhibitor of OATs, indicating the vital role of OATs. [7]. Previous studies have utilized [^14^C]-HMF which clarified that ingested HMF could be metabolized and readily eliminated during urine formation [54,56]. Intraperitoneal injection (a single dose) of 250 mg/kg SMF resulted in death of most FVB/N mice due to massive injury of the proximal tubules, while, an atypical renal hyperplasia was developed with a lower dose of SMF (125 mg/kg) [7,58]. However, additional trials are needed to observe if this hyperplasia can developed into obvious cell carcinomas [58].

### 7.3. Neurotoxic Effect

In a previous study conducted by the National Toxicology Programme (NTP) of the USA, B6C3F1 mice received 0, 188, 375 or 750 mg HMF/kg bw 5 days a week for 104 weeks. After seven months, the highest dose caused neurological symptoms such as suppression of exploratory behaviour, inability to move normally, excitation, grand mal seizures and unconsciousness [59]. On the other hand, recent studies have suggested a significant role of HMF as a novel natural antioxidant [32,60] in some neurodegenerative diseases. Different levels of HMF were used in vivo to assess their effect on apoptosis induced by H_2_O_2_ in cultured hippocampal neurons. The results showed that HMF could reduce the apoptosis of cultured hippocampal neurons [61].

### 7.4. Reproductive and Developmental Toxicity

Data from the NTP study reported that most female rats receiving 750 and 1,500 mg/kg HMF for three months had elongated estrous cycles, while the consumption of 375, 750, and 1,500 mg/kg HMF significantly increased the probability of extended diestrus [59]. In a recent study, immature female Wistar rats were given two levels of HMF (750 and 1500 mg/kg/day) for three weeks. The results showed that rats received the highest dose of HMF had earlier vaginal opening, higher luteinising hormone level, lower antimüllerian hormone level and increased number of secondary atrophic follicles [62]. These findings suggest that consumption of HMF at high levels may lead to early puberty and reduction in ovarian reserve in rats, leading to negative effects on reproductive system performance.

### 7.5. Genotoxic and Mutagenic Effect

In an in vivo study, HMF did not form any micronuclei during amicronucleus assay in marginal blood cell cultures for B6C3F1 mice that received HMF [59]. Recently, results from the Ames test conducted by Severin et al., showed a mutagenic effect of HMF at concentrations up to the IC_50_ that was not observed in bacteria. Moreover, clastogenic or aneugenic effects were also not found in a human cell line (HepG2) [63]. However, a genotoxic effect of HMF was observed by Nishi et al. [64]. Indeed, this issue is still being hotly debated. In a previous study, Surh et al. [65] reported that SMF has a potent mutagenic effect in mammalian or bacterial models in vitro. Findings from a comet assay on five cell lines showed that only at high concentrations, HMF caused clear DNA damage in all cell lines examined, however, a correlation between this damage and the activity of SULT1A1 in these cells was not observed [8]. Similar findings were assessed by the comet assay conducted by Severin et al., who reported that HMF is a DNA-damaging agent for HepG2 cells at levels from 7.87–25 mM [63]. In addition, in an in vitro study, a weak direct genotoxic and mutagenic effect of HMF was found at high molar concentrations [43]. Høie et al. [10] examined the effects of hSULT1A1/1A2 on the genotoxicity of HMF and reported that DNA from the kidneys of hSULT mice subjected to HMF treatment showed elevated damage of DNA compared with data from control hSULT animals, but no apparent changes were seen in the kidneys of wild type mice. Moreover, it was found that HMF has a potential effect to induce sister-chromatid alteration at a level of 19.8–3808.0 µM in cells exposed for 32 h [17].

### 7.6. Cytotoxicity Effect

HMF can directly react with cellular nucleophiles, while its cytotoxic doses were found to play a role in triggering mitotic dysfunction [43]. HMF at high doses is cytotoxic, evoking irritation of the upper respiratory tract, skin, eyes and mucus membranes [66]. In a 90 days gavage study conducted by NTP, cytoplasmic alteration of the kidney was significantly elevated in C3B6F1 mice received 188-, 375- and 750-mg/kg HMF [59]. Following incubation of V79 and Caco-2 cells with HMF for 1-h, moderate cytotoxicity in both cell lines was found (LC50: 115 mm and 118 mm), respectively. after 1-day of incubation, growth inhibition was obtained (V79, IC50: 6.4 mm) [43]. The comet assay on different cell lines was used in a previous study to investigate the potential of HMF to induce DNA damage, and the results showed that exposure to HMF at a concentration of 100 mM for 3 h resulted in significant DNA damage in all cell lines. At the same concentration of HMF, a modest decrease in cell viability was found in all cell lines. However, DNA damage was clearly observed in two cell lines (V79-hP-PST and V79) at three times lower concentration, suggesting a great variation among the different cell lines in sensitivity to DNA damage, but under this condition, there was no association between DNA damage and the viability of the V79 cells [8]. These findings suggest the probability of existing vital mechanisms other than cytotoxicity beyond the HMF-induced DNA damage.

### 7.7. Inhibition of Enzymes Effect

DNA polymerases (pols) play a key role in DNA repair and replication. DNA pols are the central enzymes required to maintain the integrity of the genome under the different conditions [67] since pols inhibition or their dysfunction leads to several biological dysfunctions [68]. HMF has been found to inhibit in vitro the activities of some mammalian DNA polymerases. Mizushina et al. [69] rereported that HMF has a potent and selective inhibitory action against the activities of mammalian DNA pol λ and terminal deoxynucleotidyltransferase (TdT) which are family X pols, in vitro. The authors suggested that HMF binds to the pol β-like region of pol λ and TdT.

## 8. Prooxidant Effects of HMF

Janzowski et al. [43] found that a high concentration of HMF (120 mM) caused a depletion in the concentration of glutathione (GSH) in different mammalian cultured cells. However, there was no detectable damage to DNA. Furfural is oxidized in liver into pyromucic acid which has a toxic effect on hepatocytes, however, creation of glycine in the liver helps in furfural detoxification via conjugate with pyromucic acid and excretion in urine [70,71]. However, there is a shortage of studies related to the in vivo prooxidant effects of HMF and the probability of being a mechanism of its toxic effect.

## 9. Carcinogenic Effects of HMF

It has been demonstrated that consumption of HMF in a high dose may result in the initiation of tumorigenic activities. HMF and its derivative SMF induces neoplastic transformations in different tissues. Findings from a previous study reported that HMF has the potential to induce aberrant crypt foci (ACF) in a dose-dependent manner [9,72]. However, HMF is a weak intestinal carcinogen. Moreover, some HMF derivatives such as sulfoxymethyl and chloromethyl were found to exhibit high skin tumor-initiating activity [65]. When topically applied to the skin of B6C3F1 mice, HMF showed lower induction of skin papilloma than its derivative SMF [65]. In contrast, Florian et al. reported that neither HMF nor its metabolite SMF caused ACF and intestinal carcinoma [73]. Peroral application of 188 mg HMF/kg for 104 weeks could induce hepatocellular adenomas in female B6C3F1 mice in comparison to control mice [59]. The dosage of 188 mg HMF/kg clearly elevated the prevalence of liver adenoma in female mice over a 24 month trial [59].

## 10. Conclusions

Multiple factors affect HMF formation in different food products such as thermal-treatment, duration and condition of storage and food components. However, the toxic effects of HMF depend mainly on its concentration in consumed food. Only high concentration doses have adverse effects on human and animal health. The correct evaluation of HMF formation in the different food products and the assessment of its health risks are very important and need more studies. In addition, evaluation of the novel strategies to mitigate the adverse effects of such contaminants on human health also should be taken into consideration.
